# Incidence of misophonia, associated auditory symptoms and sensory processing profiles in university students in Turkey: a comparative study of clinical, subclinical and control groups

**DOI:** 10.1017/S0022215126104526

**Published:** 2026-05

**Authors:** Gurbet İpek Şahin Kamişli, Özlem Ülkeroğlu, Sude Karataş, Aleyna Nur Durusu

**Affiliations:** Faculty of Health Sciences Audiology Department, Gazi University, Ankara, Turkey

**Keywords:** hyperacusis, sensory thresholds, tinnitus

## Abstract

**Objective:**

This study aimed to investigate the prevalence of misophonia among university students, examine its relationship with hyperacusis and tinnitus and explore the sensory processing patterns of individuals with misophonia in comparison to the control group.

**Methods:**

Based on the Misophonia Questionnaire severity scores, 81 clinical misophonia and 163 subclinical misophonia patients and 60 matched healthy-controls participated the study and evaluated with the Tinnitus Handicap Inventory, Khalfa Hyperacusis Questionnaire and the Dunn Adult/Adolescent Sensory Profile questionnaire.

**Results:**

The incidence of clinical misophonia was 19 per cent. The clinical misophonia group had significantly higher sensory-sensitivity and sensation-avoiding scores and lower low-registration scores (Mann–Whitney U-test with Bonferroni correction; *p* < 0.017). Sensory processing scores across all sensory domains were significantly correlated with Misophonia Questionnaire scores. The strongest associations were moderate positive correlations in the touch (r = 0.444; *p* = 0.00) and visual (r = 0.420; *p* = 0.00) processing domains.

**Conclusions:**

Findings indicate that misophonia involves heightened sensitivity and avoidance not only in auditory but also in movement, visual, touch and activity-related sensory areas, suggesting it is a complex atypical multisensory condition rather than purely audiological or psychiatric.

## Introduction

The concept of decreased sound tolerance is an umbrella term that includes the concepts of hyperacusis, misophonia and noise sensitivity.^1^ A complicated neurophysiological and behavioural condition with multiple underlying causes, misophonia is characterised by a heightened emotional and physiological reaction brought on by an intolerance to particular auditory stimuli.^2^ The particular sound triggers the emotional and behavioural reaction. Individuals with misophonia are generally disturbed by specific auditory stimuli, which commonly include eating-related sounds (such as chewing, smacking, slurping, or crunching), breathing, sniffing, throat sounds (e.g., throat clearing), foot tapping and machine-related noises (such as clock ticking or keyboard typing).^3^^,^^4^ These trigger sounds can vary widely between individuals. The condition often elicits a range of physiological responses, including increased heart rate, shortness of breath, elevated body temperature and heightened muscle tension.^5^ Emotional reactions may encompass anger, disgust, irritation, aggression, sweating and feelings of being overwhelmed.^6^ These responses are typically sound-induced and may lead individuals to develop avoidance behaviours as a coping mechanism.^7^

The reaction called *misophonic responses* is not related to the psychoacoustic properties of the sound, such as frequency and loudness, but is related to the individual’s past experiences and who or what is emitting the sound. Although the exact prevalence of misophonia is unknown, it is thought to be near 20 per cent of the population, with 6 per cent of those individuals exhibiting considerable functional impairment.^8^ Misophonia could be a more prevalent and underdiagnosed condition, likely due to its potential misidentification as other auditory disorders and its frequent comorbidity with psychiatric and auditory conditions.

Although misophonia has gained increasing attention in the scientific literature in recent years, the lack of clarity regarding its pathophysiology and the absence of standardised diagnostic criteria pose significant challenges for the development of specific therapeutic interventions or treatments.^9^ In an effort to facilitate diagnosis and assess the emotional and social impact as well as the severity of misophonia, various psychometric tools have been developed.^10-10^ Among the most commonly used instruments in misophonia assessment are the Amsterdam Misophonia Scale, the Misophonia Questionnaire and the Misophonia Assessment Questionnaire. From an audiological perspective, there is currently no consensus on a standardised evaluation protocol for misophonia. Hearing loss may or may not be present in affected individuals. In addition to standard audiological assessments such as pure tone audiometry and acoustic immittance testing, the evaluation of loudness discomfort levels (LDLs) is recommended.^16^

Misophonia is a complex phenomenon with both auditory and psychiatric dimensions, and it has been conceptualised as both a mental and auditory disorder. In individuals with misophonia, triggering sounds not only engage the classical auditory pathways but also activate the limbic and autonomic nervous systems, resulting in emotional, physiological and behavioural responses. Neurophysiological and neuroimaging studies have revealed heightened activity in brain regions associated with emotional processing and regulation, particularly the anterior insular cortex, amygdala and hippocampus, in individuals with misophonia. Emotional dysregulation has been identified as a core component of the condition.^6^ Psychophysiological studies evaluating autonomic nervous system function have shown that individuals with misophonia exhibit increased sympathetic nervous system responses to auditory stimuli compared to control groups. Additionally, studies have shown that people with misophonia are more likely to exhibit autistic features, and there have been reports of potential comorbidities such as panic disorder, social anxiety disorder, obsessive-compulsive disorder and certain phobias.^17^^,^^18^ These findings suggest that misophonia may involve a broad neural network within the central nervous system and may significantly affect emotional processing.

The neurological process by which the central nervous system gathers, arranges and interprets sensory data from the body and surroundings in order to produce appropriate behavioural responses, coordinate motor planning and create spatial–temporal understanding is known as sensory integration.^19^ This process enables individuals to effectively engage with their physical and social environments by synthesising and regulating incoming sensory input. Sensory data are gathered through receptors associated with various modalities—including vision, hearing, smell, taste, touch, vestibular (balance) and proprioception (body position)—and are then analysed, modulated and integrated. Through this integration, individuals develop adaptive responses essential for daily functioning.^20^

Sensory processing is defined as the brain’s ability to encode, interpret, store and retrieve sensory input in order to generate appropriate behavioural responses.^21^ Sensory processing disorder (SPD) refers to difficulties in efficiently processing sensory information, where stimuli may be under- or over-responded to, or not registered appropriately. Individuals with SPD may experience sensory hypersensitivity, sensory seeking behaviours, sensory avoidance, or sensory under-responsiveness. According to Dunn’s Sensory Processing Framework (1997), behavioural patterns arise from the interaction between neuroscientific and behavioural constructs. The neuroscientific component addresses how sensory input is detected, while the behavioural component focuses on how the individual responds to this input.^22^

Dunn’s model characterises sensory processing into four quadrants: Sensory Sensitivity, defined by a low neurological threshold with heightened responses to sensory stimuli; Sensation Avoiding, where individuals actively avoid sensory input due to low thresholds; Low Registration, characterised by high thresholds and delayed or reduced responses to stimuli; and Sensation Seeking, where individuals with high thresholds actively pursue intense sensory experiences.^22^ This model is based on the connection between a person’s behavioural response strategy and their neurological threshold. Individuals with low thresholds respond rapidly and intensely to minimal stimuli, while those with high thresholds may require stronger or more prolonged stimuli to elicit a response, and may even miss stimuli altogether.^23^ Behavioural adaptations develop based on these thresholds—low-threshold individuals may withdraw from or avoid stimuli, while high-threshold individuals may engage in sensory-seeking behaviour to meet their sensory needs.

Misophonia is known to be associated with maladaptive sensory responses, where individuals display heightened sensitivity and intolerance to specific trigger sounds, leading to hyper-reactivity and avoidance behaviours. These reactions are known to negatively affect both functional performance and overall well-being.^24^ It is suggested that such responses could be part of a broader sensory processing profile and may overlap with other auditory conditions such as tinnitus and hyperacusis.

In addition to the pronounced auditory sensitivity observed in misophonia, the avoidance behaviours and hypersensitivity reactions elicited by trigger sounds raise questions about how individuals with misophonia process and modulate stimuli in other sensory domains. Specifically, there is growing interest in understanding whether and how individuals with misophonia differ in their perception and regulation of sensory input related to taste, smell, physical activity, vision and touch.

Misophonia has been investigated in university student populations across different countries, including the United States,^25^ China^26^ and Iran.^27^ Several studies have also investigated its relationship with auditory symptoms, including tinnitus and hyperacusis. However, there is a limited number of studies examining sensory processing characteristics across other sensory modalities in individuals with misophonia. For instance, Andermane *et al.* (2023) used the Glasgow Sensory Questionnaire to assess sensory sensitivity in individuals with misophonia and found hypersensitivity in non-auditory sensory domains.^28^ Similarly, Efraim Kaufman *et al.* (2022) reported increased sensory responsiveness,^29^ while Wu *et al.* (2014) described sensory over-responsivity across multiple domains^25^ and Zhou *et al.* (2017) referred to general sensory sensitivities.^26^ Despite these findings, the detailed processing patterns and behavioural characteristics in various sensory domains remain underexplored. The present study aims to (1) investigate the incidence of misophonia among university students, (2) examine its relationship with auditory symptoms such as tinnitus and hyperacusis and (3) investigate sensory processing characteristics using self-report measures in both individuals with misophonia, a matched control group and the population norms.

## Materials and methods

The study was conducted among university students from various universities across Turkey, regardless of their field of study. A total of 499 participants aged between 18 and 26 years (mean = 20.2, SD = 1.6) were included in the first phase of the study. Participants included students who were 18 years of age or older, received their education in Turkish and whose native language was Turkish. Individuals who were unable to complete the scales and forms, had additional disabilities or had any condition preventing them from completing the questionnaires were excluded from the study. Data were collected using both online and paper-and-pencil versions of the questionnaires. This study was performed in line with the principles of the Declaration of Helsinki. Ethical approval was obtained from the University Ethics Committee (research code: 2022 – 750; approval no.: E-77082166-604.01.02-380523). Written informed consent was obtained from all participants before data collection.

### Study design and procedure

The primary aim of the study was to investigate the prevalence of misophonia and co-occurring symptoms such as tinnitus and hyperacusis, as well as to examine the sensory processing characteristics of individuals with misophonia.

In the first stage of data collection, participants were asked a screening question:

“Are you disturbed or triggered by specific environmental sounds such as smacking, slurping, throat clearing, fork/knife scratching a plate, or a ticking clock, which are usually unnoticed by others?” Participants who answered “yes” to this question (*N* = 244) were identified as “subjective misophonia”. Based on their scores on the misophonia severity score in the Misophonia Questionnaire (MQ), this group was further divided into clinical (≥ 7) and subclinical (< 7) misophonia groups. From those who answered “no” to the initial screening question, 60 individuals were randomly selected to form the control group for further analysis.

In the second stage, several standardised scales and questionnaires were administered to clinical misophonia, subclinical misophonia and control groups. To minimise participant fatigue and ensure data reliability, a flexible timeframe was provided for completion of the forms. Participants had the option to complete the assessments either at home or online.

### Data collection tools

A structured interview form was used to collect demographic data, general health history, noise exposure, hearing health and the presence of auditory symptoms such as misophonia, hyperacusis and tinnitus. Subsequently, validated scales were administered to assess: severity of misophonia (MQ), symptoms of tinnitus and hyperacusis, sensory processing and sensory integration profiles.

*Tinnitus:* Tinnitus was assessed using the Tinnitus Handicap Inventory (THI). The THI evaluates the impact of tinnitus on quality of life through its functional, emotional and catastrophic subscales, providing information on the severity and level of tinnitus. The inventory consists of 25 items divided into three dimensions: functional, emotional and catastrophic. Responses are scored as 0–2–4, with “No” responses scoring 0, “Sometimes” responses scoring 2 and “Yes” responses scoring 4. The total score is calculated to determine the level of tinnitus, with higher scores indicating greater severity. The total score ranges from 0 to 100.^30^ Participants with a THI score of 18 or above (level 2 and above) were included in the study.

*Hyperacusis:* Hyperacusis was evaluated using the Khalfa Hyperacusis Questionnaire (KHQ). This instrument consists of 14 items rated on a 4-point Likert scale and includes three subscales: attentional, social and emotional. Response options are scored as follows: “No” (0 points), “Yes, a little” (1 point), “Yes, quite a lot” (2 points) and “Yes, a lot” (3 points). The total score is obtained by summing the item scores, with a maximum possible score of 42.^31^ A total score of 28 or above is interpreted as indicative of hyperacusis. The Turkish version of the scale was validated for reliability and validity by Erinc *et al.*^32^

*Misophonia:* Misophonia was assessed using the MQ,^10^^,^^25^ which measures misophonia symptoms as well as emotional and behavioural responses to trigger sounds. Items are rated on a 5-point Likert scale ranging from “Never” (0) to “Always” (4), and total scores range from 0 to 68. The questionnaire’s fourth section, which is not part of the factor structure or scoring, offers details on the degree of misophonia. In this section, participants are asked to rate the number and intensity of trigger sounds and the extent to which these sounds interfere with their daily life, using a scale ranging from 1 to 15. A score of 7 or higher in this section is considered clinically significant for misophonia. Higher total scores indicate greater frequency of misophonia symptoms and an increase in negative emotional and behavioural reactions to trigger sounds.

*Sensory processing:* Sensory processing was assessed using the Dunn Adult/Adolescent Sensory Profile (DASP) questionnaire.^33^ This tool assesses people’s preferences and daily sensory processing patterns based on Dunn’s sensory processing model.^34^ The model distinguishes between two core aspects: the neuroscientific component, which addresses how the nervous system detects and responds to sensory stimuli, and the behavioural component, which concerns how individuals adapt to or manage sensory input. The six sensory domains of Taste/Smell, Movement, Visual, Touch, Auditory Processing and Activity Level comprise the 60 items that consist the DASP. Participants’ responses are assessed across four sensory processing quadrants: Low Registration, Sensory Sensitivity, Sensation Avoiding and Sensation seeking. Each quadrant includes 15 items. Responses are rated on a 5-point Likert scale ranging from “Almost never” to “Almost always.” The total score for each quadrant is calculated by summing the scores of the relevant items. Normative data for the Turkish population have been established for different age groups, allowing classification of sensory processing patterns. For the 18–64 age group, the normative score ranges considered “similar to most people” are as follows: Low Registration (24–35), Sensation Seeking (43–56), Sensory Sensitivity (26–41) and Sensation avoiding (27–41). Scores falling below or above these ranges are interpreted as “less or much less than most people” and “more or much more than most people,” respectively. In the present study, DASP data were analysed both by comparing them with the Turkish normative values and by conducting between-group comparisons. Additionally, the auditory processing subsection of the DASP was examined separately to evaluate differences in auditory sensory processing among the groups.

### Statistical analysis

SPSS Statistics v.23.0 was used for all statistical analyses. Using analytical techniques and histogram graphics, the data’s normal distribution was investigated. For properly distributed data, the descriptive statistics were displayed as mean and standard deviation; for non-normally distributed data, they were displayed as median and range. The Kruskal–Wallis test was used to assess the group comparisons. The significance of pairwise differences was assessed using a Mann–Whitney U test with Bonferroni adjustment (post hoc test) to account for multiple comparisons. A *p* value less than 0.017 (0.05/3) was deemed statistically significant using the Bonferroni correction. The correlation between misophonia and adolescent/adult sensory profile scores, as well as tinnitus and misophonia, was tested with Spearman’s correlation analyses. Frequencies and percentages were used to summarise categorical variables. 0.05 was used as the statistical significance level.

## Results

In the first phase of the study, 499 university students were included to subjectively determine the incidence of misophonia. Participants were asked the question: “Are you disturbed or triggered by specific environmental sounds such as smacking, slurping, throat clearing, fork/knife scratching a plate, or a ticking clock sounds that typically go unnoticed by others?” A total of 244 students (48.8%) reported being sensitive to at least one specific sound, indicating subjective misophonia.

The 244 participants with subjective misophonia were then classified into two groups based on their MQ severity score: clinical misophonia (score ≥7) and subclinical misophonia (score <7). Of these participants, 163 were categorised as having subclinical misophonia and 81 as having clinical misophonia. This means that among those who reported misophonia symptoms, 26.6 per cent were in the clinical group and 53.6 per cent in the subclinical group. Additionally, 76 participants (25%) reported being disturbed by loud sounds, suggesting the presence of subjective hyperacusis, and 58 students (19.1%) reported experiencing tinnitus.

Information regarding participants’ hearing history, and noise exposure is presented in Table 1. Significant differences were observed between groups in terms of hyperacusis and noise exposure. Among those who reported noise exposure, 55 per cent were in the clinical misophonia group. No significant differences were found between groups in terms of hearing loss, history of ear surgery, tinnitus or psychiatric disorders (chi-squared test; *p* > 0.005). A total of 30 participants reported having a psychiatric diagnosis: 9 individuals in the control group had anxiety disorders; in the subclinical misophonia group, 8 reported anxiety, 2 attention-deficit hyperactivity disorder (ADHD) and 3 depression; in the clinical misophonia group, 1 had bipolar disorder, 3 depression, 2 obsessive–compulsive disorder (OCD).

**Table 1.** Characteristics of the groups Table 1 long description.

		Control (*n* = 60)	Subclinical misophonia (*n* = 163)	Clinical misophonia (*n* = 81)	Total
Parameter		n (%)	n (%)	n (%)	n (%)
Gender	Male	25 (8.2%)	67 (22%)	36 (11.8%)	128 (42.1%)
	Female	35 (11.5%)	96 (31.6%)	45 (14.8%)	176 (57.9%)
Hearing loss		0	1 (0.3%)	2 (0.7%)	3 (1%)
Ear surgery		0	2 (0.7%)	2 (0.7%)	4 (1.3%)
Tinnitus		6 (2%)	33 (10.9%)	19 (6.3%)	58 (19.1%)
Hyperacusis		2 (0.7%)	43 (14.1%)	31 (10.2%)	76 (25%)
Psychiatric diagnosis		9 (3%)	13 (4.3%)	8 (2.6%)	30 (9.9%)
Noise exposure		7 (2.3%)	29 (9.5%)	26 (8.6%)	62 (20.4%)

The mean age was 20.4 ± 1.7 (min. = 18, max. = 26) years in the control group (*N* = 60), 20.2 ± 1.7 (min. = 18, max. = 26) years in the subclinical misophonia group (*N* = 163) and 20.3 ± 1.3 (min. = 18, max. = 24) years in the clinical misophonia group (*N* = 81). THI, KHQ and MQ scores were compared between the groups, and a statistically significant difference was observed. (Kruskal–Wallis test, *p* < 0.05). Post-hoc pairwise comparisons using the Bonferroni correction revealed significant differences between all groups (Mann–Whitney U-test with Bonferroni correction, *p* < 0.017) (Table 3). The control group had the lowest scores for tinnitus, misophonia and hyperacusis, while the clinical misophonia group had the highest.

DASP scores, covering the four sensory quadrants (Low Registration, Sensation Seeking, Sensory Sensitivity and Sensation Avoiding) as well as sensory domains including Auditory Processing, Taste/Smell Processing, Movement Processing, Visual Processing, Touch Processing and Activity Level, were compared across groups and presented in Tables 2 and 3 (Kruskal–Wallis test, *p* < 0.05). A statistically significant difference was found across all groups in the Sensory Avoiding domain, with the clinical misophonia group showing significantly higher scores (Mann–Whitney U-test with Bonferroni correction, *p* < 0.017). No significant difference was observed in Sensory Sensitivity scores between the control and subclinical misophonia groups; however, significant differences were identified between the control and clinical, as well as between subclinical and clinical misophonia groups (*p* < 0.017). The clinical misophonia group exhibited the highest sensory sensitivity scores. No significant differences were observed in pairwise comparisons in the Sensation Seeking domain. For Low Registration, significant differences were found between the subclinical and clinical groups and between the control and clinical groups (Table 3).

**Table 2.** Evaluation scores of the groups Table 2 long description.

Parameter		Control Mean ±SD	Subclinical misophonia Mean ±SD	Clinical misophonia Mean ±SD	Total Mean ±SD
Misophonia	MQ total score	3.8±3.3	23.3±9.9	48.8±6.4	26.2±16.3
	MQ-MS	2.3±2.1	11.3±5.5	21.2±4.6	12.2±8.0
	MEB-AI	1.2±1.6	8.4±3.6	16.9±3.8	9.20±6.3
	MEB-AE	0.2±0.8	4.0±2.9	10.4±3.4	5..0±04.5
Tinnitus	THI	1.1±3.1	8.6±15.9	20.4±23.8	10.3±18.2
Hyperacusis	KHQ	6.5±4.9	11.8±6.5	17.6±9.8	12.3±8.2
DASP quadrants	Low Registration	28.9±12.5	26.7±8.2	24.3±4.3	26.5±8.6
	Sensation Seeking	33.5±9.1	31.2±10.0	29.5±7.9	31.2±9.3
	Sensory Sensitivity	28.8±10.5	31.2±10.5	36.3±11.0	32.1±10.9
	Sensation Avoiding	26.4±10.5	30.6±10.3	36.8±10.4	31.4±11.0
Scores of sensory processing categories	Auditory Processing	22.2±6.9	25.0±7.4	28.1±6.7	25.3±7.4
	Taste/Smell Processing	22.1±3.2	22.1±3.7	23.7±4.5	22.5±3.9
	Movement Processing	18.6±3.2	19.7±4.4.	22.0±5.5	20.1±4.7
	Visual Processing	23.6±4.7	25.7±4.6	29.0±5.4	26.2±5.2
	Touch Processing	27.9±5.5	31.2±6.0	35.9±6.2	31.8±6.6
	Activity Level	24.0±5.8	25.4±4.8	28.8±5.1	26.0±5.4

DASP = Dunn Adult/Adolescent Sensory Profile; MQ = Misophonia Questionnaire; MQ-MS = Misophonia Questionnaire Misophonia Symptoms; MEB-AI = Misophonia Emotions and Behaviors-Avoidance and Internalization; MEB-AE = Misophonia Emotions and Behaviors-Aggression and Externalization; THI = Tinnitus Handicap Inventory; KHQ = Khalfa Hyperacusis Questionnaire.

**Table 3.** Pairwise comparison of THI, KHQ, DASP scores between groups Table 3 long description.

Variables		Control–subclinic misophonia, *p* value	Control-clinic misophonia, *p* value	Subclinic-clinic misophonia, *p* value
	MQ	0.000*	0.000*	0.000*
	MQ-MS	0.000*	0.000*	0.000*
	MEB-AI	0.000*	0.000*	0.000*
	MEB-AE	0.000*	0.000*	0.000*
	THI	0.002*	0.000*	0.000*
	KHQ	0.000*	0.000*	0.000*
DASP quadrants	Low Registration	.400	0.001*	0.014*
	Sensation Seeking	.265	0.037	0.644
	Sensory Sensitivity	.447	0.000*	0.004*
	Sensation Avoiding	.011*	0.000*	0.001*
Sensory areas	Auditory Processing	.025	0.000*	0.004*
	Taste/Smell Processing	1.000	0.030	0.029
	Movement Processing	.183	0.000*	0.003
	Visual Processing	0.019	0.000*	0.000*
	Touch Processing	0.002*	0.000*	0.000*
	Activity Level	0.518	0.000*	0.000*

*Note:* Kruskal–Wallis test used for the comparison of the groups. Pairwise comparisons tested with Mann–Whitney U-test with Bonferroni correction (signification level *p* < 0.017 [0.05/3]).

MQ = Misophonia Questionnaire; MQ-MS = Misophonia Questionnaire-Misophonia Symptoms; MEB-AI = Misophonia Emotions and Behaviors-Avoidance and Internalization; MEB-AE = Misophonia Emotions and Behaviors-Aggression and Externalization; THI = Tinnnitus Handicap Inventory; KHQ = Khalfa Hyperacusis Questionnaire.

Regarding sensory processing subdomains, the clinical misophonia group had higher scores in all areas Auditory Processing, Taste/Smell Processing, Movement Processing, Visual Processing, Touch Processing and Activity Level (Kruskal–Wallis test; *p* < 0.05). Pairwise comparison tests revealed that Touch Processing scores differed significantly between all groups. In the Activity Level, Visual Processing and Auditory Processing domains, no significant differences were observed between the control and subclinical groups; however, statistically significant differences were found when comparing the control vs. clinical and subclinical vs. clinical misophonia groups (Mann–Whitney U-test; *p* < 0.017) (Table 3).

Table 4 presents the correlation findings between misophonia scores and other variables. Both the total misophonia score and the misophonia domains (MQ-MS, MEB-AI, MEB-AE) Spearman’s correlation test showed statistically significant moderate correlation with THI (r = 0.371, r = 0.365, r = 0.308, r = 0.324, *p* < 0.05), statistically significant moderate correlation with KHQ (r = 0.464, r = 0.366, r = 0.485, r = 0.416, *p* < 0.05), statistically significant moderate correlation with Sensory Sensitivity (r = 0.244, r = 0.204, r = 0.213, r = 0.251, *p* < 0.05) and Sensation Avoiding (r = 0.316, r = 0.277, r = 0.295, r = 0.315, *p* < 0.05). Notably, Sensation Seeking (r = -0.197, r = -.0183, r = -0.195, r = -0.135, *p* < 0.05) and Low Registration (r = -0.177, r = -0.149, r = -0.170, r = -0.158, *p* < 0.05) scores have a weak negative correlation with total and domain-specific misophonia scores (Spearman’s correlation test, *p* < 0.05). As seen in Table 4, significant correlations were observed between all sensory domains and misophonia scores, with the strongest correlations identified with a positive moderate correlation in the touch (r = 0.444; *p* = 0.00) and visual processing domains (r = 0.420; *p* = 0.00).

**Table 4.** Correlations between misophonia and adolescent/adult sensory profile scores Table 4 long description.

		MQ total score	MQ-MS	MEB-AI	MEB-AE
THI	Correlation coefficient	0.371	0.365	0.308	0.324
	Sig. (2-tailed)	0.00*	0.00*	0.00*	0.00*
KHQ	Correlation coefficient	0.464	0.366	0.485	0.416
	Sig. (2-tailed)	0.00*	0.00*	0.00*	0.00*
Low Registration	Correlation coefficient	−0.177	−0.149	−0.170	−0.158
	Sig. (2-tailed)	0.002*	0.009*	0.003*	0.006*
Sensation Seeking	Correlation coefficient	−0.197	-.0183	−0.195	−0.135
	Sig. (2-tailed)	0.001*	0.001*	0.001*	0.018*
Sensory Sensitivity	Correlation coefficient	0.244	0.204	0.213	0.251
	Sig. (2-tailed)	0.001*	0.000*	0.00*	0.000*
Sensation Avoiding	Correlation coefficient	0.316	0.277	0.295	0.315
	Sig. (2-tailed)	0.00*	0.000*	0.000*	0.000*
Auditory	Correlation coefficient	0.349	0.280	0.354	0.273
	Sig. (2-tailed)	0.000*	0.000*	0.000	0.000
Taste/Smell	Correlation coefficient	0.210	0.213	0.200	0.097
	Sig. (2-tailed)	0.00*	0.00*	0.00*	0.91
Movement	Correlation coefficient	0.264	0.275	0.244	0.197
	Sig. (2-tailed)	0.00*	0.00*	0.00*	0.001*
Visual	Correlation coefficient	0.395	0.312	0.420	0.334
	Sig. (2-tailed)	0.00*	0.00*	0.00*	0.00*
Touch	Correlation coefficient	0.444	0.409	0.398	0.387
	Sig. (2-tailed)	0.00*	0.00*	0.00*	0.00*
Activity Level	Correlation coefficient	0.367	0.312	0.374	0.299
	Sig. (2-tailed)	0.00*	0.00*	0.00*	0.00*

* p: Statistically significant *p* < 0.05, Spearman’s correlation test.

MQ = Misophonia questionnaire; MQ-MS = misophonia Questionnaire misophonia symptoms; MEB-AI = Misophonia Emotions and Behaviors-Avoidance and Internalization; MEB-AE = Misophonia Emotions and Behaviors-Aggression and Externalization; THI = Tinnnitus Handicap Inventory; KHQ = Khalfa Hyperacusis Questionnaire.

As shown in Table 5, the distribution of participants across sensory processing patterns varied by group. Within the clinical misophonia group, 61.7 per cent were categorised as “Much less than most people” (*n* = 30) and “Less than most people” (*n* = 20) in the Sensation Seeking pattern. In contrast, 28.3 per cent of this group were categorised as “More than most people” (21) and “Much more than most people”(*n* = 2) in the Sensation Avoiding pattern, while 40.7 per cent (*n* = 38) fell into the same upper categories for the Sensory Sensitivity pattern.

**Table 5.** Distribution of the groups according to the four quadrants of DASP Table 5 long description.

	Much less than most people			Less than most people			Similar to most people			More than most people			Much more than most people		
	N			N			N			N			N		
Quadrant	Control	Subclinical	Clinical	Control	Subclinical	Clinical	Control	Subclinical	Clinical	Control	Subclinical	Clinical	Control	Subclinical	Clinical
Low Registration	14	12	1	8	31	7	23	62	34	7	41	19	8	17	20
Sensation Seeking	11	51	30	15	54	20	23	58	27	10	0	3	1	0	1
Sensory Sensitivity	0	10	1	14	44	10	32	80	32	13	21	27	1	8	11
Sensation Avoiding	13	12	5	9	32	20	27	77	33	11	36	21	0	6	2

## Discussion

In this study, we investigated the prevalence of misophonia among university students, its association with auditory symptoms such as hyperacusis and tinnitus, and the sensory processing characteristics of individuals with misophonia. The study included 499 young adults and relied on self-report data. According to responses collected through the interview form, 244 students (48.8%) reported subjective misophonia symptoms. Among those with misophonia, 58 students reported experiencing tinnitus and 76 reported hyperacusis. The literature indicates that the prevalence of misophonia varies widely in this age group. Studies conducted with undergraduate university students have reported self-reported misophonia symptoms in 23 per cent of participants in Iran,^27^ 49 per cent in the UK^35^ and approximately half of the participants in Serbia^36^ and Turkey.^10^ Similarly, studies from the USA (23.4%) and India (48%)^37^ have reported notable prevalence rates. However, these prevalence rates tend to decrease when misophonia is assessed using specific diagnostic tools or when clinical misophonia is considered. For example, Sarıgedik *et al.*^38^ reported a prevalence of 13 per cent for moderate or severe misophonia in a Turkish sample, while Deniz Sakarya and Çakmak identified clinical misophonia in 19 per cent of their sample of 84 individuals. In other studies, Pfeiffer *et al*. reported a prevalence of 21 per cent,^39^ Zhou *et al*. 6 per cent,^26^ and Brennan *et al.* 20 per cent^8^ for clinical misophonia. In our study, 19.2 per cent of participants were classified within the clinical misophonia group. These findings are consistent with previous research conducted in Turkey, as well as studies from Serbia, the UK and India.

In this study, participants were divided into two groups based on the severity score of the MQ: clinical misophonia (≥ 7) and subclinical misophonia (< 7). The mean total MQ score in the clinical misophonia group was found to be 48.8 ± 6.4. In a study conducted among university students in China, the mean MQ score for a clinical misophonia group of 33 participants was 33.1 ± 10.7.^26^ Similarly, Wu *et al.* reported a mean score of 31.2 in a U.S.-based study.^25^ A previous study conducted in Turkey among university students reported a mean score of 40.7.^10^ Both the prevalence of misophonia and the average MQ scores were found to be higher in our sample.

Misophonia has been associated with other mental health conditions.^40^ Mental health is influenced by various factors, including biological, social, developmental and political aspects, as well as the environment, living conditions and how individuals cope with stressors. According to the World Health Organization (WHO), mental health challenges are increasingly prevalent in Turkey. Approximately 20 per cent of the population is reported to experience psychological conditions that require treatment, and the use of psychiatric medication has also increased in recent years.^41^ In line with this information, the higher prevalence and severity of misophonia observed in our study may be partially explained by the broader mental health landscape in Turkey.

Misophonia has been linked to several psychiatric disorders, including specific phobia, post-traumatic stress disorder, OCD and social anxiety disorder.^25^^,^^26^^,^^40^ In our sample, 21 participants (7.9%) who exhibited misophonia symptoms had a diagnosed psychiatric condition (Table 1). These included primarily depression and anxiety, as well as bipolar disorder, obsessive-compulsive disorder, social phobia, panic attacks and ADHD.

As shown in Table 1, only 1 per cent of participants reported having hearing loss. While misophonia is an auditory symptom, it has not been found to be associated with hearing loss.^8^^,^^40^ On the other hand, participants showed similar distributions across groups in terms of history of ear surgery and hearing loss. Noise exposure is a known risk factor for hearing loss and various auditory complaints. In our sample, individuals with greater noise exposure were more likely to be in the misophonia groups.

Hyperacusis and tinnitus were assessed both through standardised scales and direct self-report via the interview form. Among participants exhibiting misophonia symptoms, 52 individuals (21.3%) reported experiencing tinnitus and 74 individuals (30.3%) reported hyperacusis. Within the clinical misophonia group (*n* = 81), 31 participants (38%) reported hyperacusis and 19 (23.4%) reported tinnitus. In this study, when assessed using validated scales, the mean score on the KHQ in the clinical misophonia group was 17.6, and the mean score on the THI was 20.4. Pairwise comparisons between all groups revealed statistically significant differences (Table 3). Both hyperacusis and tinnitus levels were found to be highest in the clinical misophonia group. Similarly, Brennan *et al.* reported higher KHQ scores in individuals with misophonia within a comparable age group and found a strong correlation between KHQ and HQ scores. In the same study, individuals with self-reported tinnitus were also found to have higher MQ scores.^8^ Consistent with the findings of Brennan *et al.*, our study also showed that misophonia was more frequently comorbid with hyperacusis than with tinnitus.

Misophonia is not solely an auditory symptom; it involves a strong connection between the auditory pathways and the limbic and autonomic nervous systems, which becomes activated in response to trigger sounds.^42^ Exposure to such sounds has been shown to increase heart rate and galvanic skin response, indicating activation of the anterior insular cortex. Eijsker *et al.* found both structural and functional brain abnormalities in individuals with misophonia. These included larger right amygdala volumes, stronger resting-state coupling between the amygdala and cerebellum, and increased connectivity of the ventral attention network within the occipital cortex.^43^

Individuals with misophonia exhibit patterns of increased sympathetic activation in response to trigger sounds. Neuroimaging studies have demonstrated the involvement of multiple brain regions in misophonia, including the temporal cortex, hippocampus, amygdala (a key limbic structure), the ventromedial prefrontal, premotor and cingulate cortices as well as the cerebellum. When exposed to trigger sounds, hyperactivity has been observed not only in the bilateral auditory cortex but also in the left amygdala. Amygdala enlargement and increased activity in attentional networks may explain emotional and attentional abnormalities in these individuals.^44^

Kumar *et al.* identified increased neural activity in the anterior insular cortex of individuals with misophonia.^45^ The insular cortex is known to play a role in auditory processing, nonverbal stimuli perception and phonological processing.^46^ It is anatomically adjacent to the primary auditory cortex and is involved in autonomic control, memory, mood regulation, olfactory and gustatory perception, interoceptive awareness and social cognition. It serves as a central hub for interoceptive signals and emotional processing.^47^ These neurobiological findings may explain the emotional and behavioural responses—particularly their intensity—seen in individuals with misophonia when exposed to trigger sounds.

The hypersensitivity and avoidance responses to specific auditory stimuli observed in individuals with misophonia raise the question of whether similar sensitivities or sensory processing issues exist in other sensory domains. Given the involvement of such diverse brain regions—particularly those responsible for sensory regulation—this question appears justified.

Sensory processing disorder (SPD) broadly refers to difficulties in the brain’s ability to respond appropriately to sensory input, manifesting as either hypersensitivity or hyposensitivity to ordinary stimuli—i.e., neurologically atypical responses. In the present study, we examined sensory integration and processing in individuals exhibiting misophonia symptoms. Sensory domains assessed included Taste/Smell, Movement, Vision, Touch, Auditory Processing and Activity Level. Responses to sensory input (reflecting neurological thresholds and behavioural responses) were categorised as Low Registration, Sensation Seeking, Sensation Avoiding and Sensory Sensitivity.

The clinical misophonia group exhibited the highest scores in both Sensation Avoiding and Sensory Sensitivity categories. Pairwise group comparisons and correlation analyses indicated that increases in misophonia symptom severity were associated with increases in Sensation Avoiding and Sensory Sensitivity. According to Dunn’s model, which posits the intersection of neurological thresholds and behavioural responses, these findings suggest that individuals with misophonia have low neurological thresholds and cope by avoiding sensory stimuli.

In all sensory domains except taste/smell processing, the clinical misophonia group had higher scores than the control group. Mulligan *et al.* noted that individuals with sensory processing disorders (SPD) often struggle with auditory modulation.^48^ Sensory modulation disorder (SMD), a subtype of SPD, refers specifically to difficulties in regulating responses to sensory input. It includes three subtypes: sensory over-responsivity, sensory under-responsivity and sensation seeking and encompasses all sensory systems, including interoception.

The capacity to recognise, control and react correctly to everyday sensory stimulation is known as sensory modulation. Individuals with misophonia often display auditory over-responsivity, hypersensitivity and sometimes avoidance behaviours in response to trigger sounds. The Neural Foundations of Ayres Sensory Integration® suggests that individuals with poor sensory modulation may struggle with self-regulation, attention shifting and sensory integration functions.^49^ Hyperreactivity to sensory input activates the sympathetic nervous system, suggesting a shared mechanism between misophonia and SMD.

Just as individuals with sensory modulation disorder are unable to disregard redundant or irrelevant stimuli, those with misophonia cannot ignore trigger sounds. Functional MRI (fMRI) studies in individuals with sensory modulation disorder have shown hyperactivity in the insular cortex, anterior cingulate cortex and amygdala—regions involved in assigning salience and directing attention to environmental stimuli. Due to heightened activity in these areas, these individuals tend to give excessive attention to basic sensory input,^49^ a pattern also observed in misophonia. Sensory modulation plays a critical role in filtering and prioritising the overwhelming number of stimuli we encounter from our environment.

In the clinical misophonia group, significantly higher scores were observed in the domains of Sensory Sensitivity, Sensation Avoiding and Low Registration (Table 4). These findings indicate that individuals with clinical levels of misophonia exhibit not only auditory sensitivity and avoidance, but also heightened reactivity and avoidance behaviours across other sensory domains. Our results suggest that, similar to their avoidance of trigger sounds, individuals with misophonia also tend to avoid other sensory stimuli.

Lower scores were recorded in the sensation seeking domain, suggesting that individuals with clinical misophonia are less inclined to seek out sensory stimuli, not only in the auditory domain but across multiple sensory modalities. These findings are consistent with a study conducted by Wu *et al.*, who used the Adult Sensory Questionnaire (ASQ) with a similar sample and reported significant differences between clinical and subclinical misophonia groups. They also found a significant correlation between ASQ and MQ scores. However, sensory integration was not assessed in that study, despite being one of the few studies evaluating the sensory profile of young individuals with misophonia. The authors proposed a strong relationship between misophonia and general sensory sensitivities^25^

Similarly, Zhou *et al.*, in their study of Chinese college students, found significant group differences and correlations between MQ and ASQ scores across clinical and subclinical misophonia groups. Although their study did not include a control group, they reported that both misophonia groups had higher ASQ scores compared to previously published data from Chinese and American college student populations.^26^

Consistent with these findings, our study also revealed that the clinical misophonia group had higher scores in the Avoiding and Sensitivity quadrants of the DASP compared to both the control and subclinical misophonia groups. The sensory over-responsivity (SOR) pattern observed in individuals with misophonia aligns with previous reports indicating that SOR is present in 5–15 per cent of the general population but is much more common—exceeding 50 per cent—in individuals with both genetically and environmentally based psychiatric and neurodevelopmental disorders, including anxiety, ADHD, early life adversity and autism spectrum disorder (ASD).^26^

Correlation analyses revealed significant relationships between total MQ scores and subscale scores with various sensory processing domains. Specifically, negative correlations were found between MQ scores and the Low Registration and Sensation Seeking quadrants, whereas positive correlations were observed with Sensory Sensitivity and Sensation Avoiding scores. In addition, significant correlations were identified between MQ scores and specific sensory modalities, including Auditory, Taste/Smell, Movement, Visual, Touch and Activity Level domains.

Although the clinical misophonia group exhibited higher scores, DASP quadrant scores across all groups generally fell within the range defined as “similar to most people” for this age group. This suggests that, while individuals in the clinical misophonia group demonstrated elevated scores, their sensory processing profiles were not drastically different from those observed in the general population.

However, as shown in Table 5, a notable number of individuals in the clinical misophonia group reported elevated responses in specific quadrants. Specifically, 33 participants were categorised as “more than” or “much more than most people” in the Avoiding quadrant, and 38 participants fell into the same categories in the Sensitivity quadrant. Conversely, in the Sensation Seeking quadrant, 37 individuals were categorised as “much less than most people.”

Correlation analyses revealed significant relationships between the MQ total and subscale scores and various sensory processing patterns. Specifically, MQ scores were negatively correlated with the Low Registration and Sensation Seeking quadrants and positively correlated with Sensory Sensitivity and Sensation Avoiding scores. In addition, significant correlations were observed between MQ scores and specific sensory domains, including Auditory, Taste/Smell, Movement, Visual, Touch and Activity Level. Although the clinical misophonia group showed higher mean scores, the overall DASP scores across all groups generally fell within the “similar to most people” range for the relevant age group. This indicates that, while individuals in the clinical group exhibited elevated sensory processing scores, their sensory profiles were not drastically different from the general population. However, as shown in Table 5, a more nuanced picture emerges when examining specific quadrants. Within the clinical misophonia group, 33 individuals were classified in the “more than” or “much more than most people” categories in the Sensation Avoiding quadrant and 38 individuals in the Sensory Sensitivity quadrant. Furthermore, 37 individuals were categorised as “much less than most people” in the Sensation Seeking quadrant. These findings suggest that, although mean scores may fall within normative ranges, a substantial proportion of individuals with clinical misophonia demonstrate heightened sensitivity and avoidance behaviours, alongside reduced sensory seeking tendencies.

- Individuals with clinical misophonia have sensory processing problems, especially in the sensory avoidance and sensory sensitivity areas
- Misophonia scores have positive correlations, especially in the touch and visual processing domains
- This study suggests that misophonia may not be solely an audiological or psychiatric condition, but rather a complex phenomenon that involves atypical multisensory processing.
- This study highlights the importance of adopting a multidisciplinary approach and utilising comprehensive assessment strategies in both clinical settings and future research to gain a deeper understanding of the multifaceted nature of misophonia

## Limitations

This study has several limitations. First, the assessment of misophonia and related auditory and sensory features was based entirely on self-report measures. Future studies would benefit from incorporating clinical interviews and diagnostic evaluations in collaboration with psychiatry professionals to improve diagnostic accuracy. Additionally, sensory integration and processing were also assessed using self-report tools; the inclusion of observational or performance-based assessments in future research could yield more nuanced insights. Although our findings indicate heightened sensitivity in various sensory domains, further investigation is needed to determine the effectiveness of sensory integration therapies for individuals with misophonia. Moreover, the study sample was limited to a narrow age range of university students, which may restrict the generalisability of the results. Expanding research to include a broader age range of adults would enhance the applicability of the findings.

## Conclusions

This study provided a detailed investigation of the prevalence of misophonia and associated auditory symptoms among undergraduate students, as well as the sensory processing characteristics of individuals with clinical misophonia. We found that higher misophonia scores were significantly associated with increased Sensation Avoiding and Sensory Sensitivity, alongside reduced Sensory Seeking behaviours. In our sample of 499 individuals, 19 per cent met the criteria for clinical misophonia. This group also exhibited higher rates of comorbid hyperacusis and tinnitus.

Our findings suggest that misophonia may not solely be an audiological or psychiatric condition, but one that potentially involves multisensory processing differences. Therefore, a multidisciplinary approach and comprehensive assessment strategy should be considered in both clinical practice and future research to fully understand the complexity of misophonia.

## Data Availability

The data supporting this study’s findings are available from the corresponding author upon reasonable request.

## Table 1 Long description

The table compares characteristics of control, subclinical misophonia, and clinical misophonia groups, focusing on gender, hearing loss, ear surgery, tinnitus, hyperacusis, psychiatric diagnosis, and noise exposure. Females constitute 57.9% of the total sample, with the highest percentage in the subclinical group. Clinical misophonia has the highest rates of hyperacusis (10.2%) and tinnitus (6.3%) compared to other groups. Hearing loss and ear surgery are rare across all groups, with a total occurrence of 1% and 1.3%, respectively. Psychiatric diagnosis is most common in the subclinical group (4.3%). Noise exposure is notably higher in the clinical misophonia group (8.6%) than in the control group (2.3%).

Navigate back to Table 1.

## Table 3 Long description

The table compares THI, KHQ, and DASP scores between control, subclinic, and clinic misophonia groups. Significant differences are observed in MQ, MQ-MS, MEB-AI, MEB-AE, THI, and KHQ scores across all group comparisons, indicating strong misophonia symptoms. Sensory processing areas show varied significance, with auditory, visual, and touch processing having significant differences between control and clinic groups. Sensation Avoiding is notably significant across all comparisons, while Sensation Seeking shows less variation. The Kruskal–Wallis test and Mann–Whitney U-test with Bonferroni correction were used, suggesting robust statistical analysis.

Navigate back to Table 3.

## Table 2 Long description

The table evaluates sensory and emotional processing scores across control, subclinical, and clinical misophonia groups. Clinical misophonia consistently shows higher scores in misophonia symptoms, emotional behaviors, tinnitus, and hyperacusis compared to other groups. Sensory sensitivity and sensation avoiding are notably higher in the clinical group, indicating increased sensory processing challenges. Auditory, visual, touch, and activity level processing scores are also elevated in clinical misophonia, suggesting broader sensory processing difficulties. Subclinical misophonia scores are intermediate, while control scores are lowest across all parameters. These trends highlight the severity of sensory and emotional processing issues in clinical misophonia.

Navigate back to Table 2.

## Table 4 Long description

The table measures correlations between misophonia and sensory profile scores in adolescents and adults. KHQ scores have the highest positive correlation with misophonia symptoms, particularly with MEB-AI (0.485) and MQ total score (0.464). Sensation seeking shows a negative correlation across all misophonia measures, with the strongest negative correlation for MQ total score (-0.197). Sensory sensitivity and sensation avoiding also show positive correlations, indicating that higher sensitivity and avoidance are associated with increased misophonia symptoms. The correlations are statistically significant, with p-values less than 0.05, except for taste/smell with MEB-AE, which is not significant. These findings suggest that sensory processing patterns are linked to misophonia severity.

Navigate back to Table 4.

## Table 5 Long description

The table measures the distribution of groups across four quadrants of DASP: Low Registration, Sensation Seeking, Sensory Sensitivity, and Sensation Avoiding. Subclinical groups consistently have higher numbers in sensation seeking and sensation avoiding, particularly in the 'Less than most people' and 'Similar to most people' categories. Control groups generally have lower numbers across all quadrants, except for sensation seeking where they have a notable presence in the 'Similar to most people' category. Clinical groups show lower numbers overall, with some presence in sensation avoiding. The data suggests that subclinical groups may have heightened sensory processing compared to control and clinical groups, but further analysis is needed to understand the implications fully.

Navigate back to Table 5.

## References

[1] Aazh H, Kula FB. The Sound Sensitivity Symptoms Questionnaire Version 2.0 (SSSQ_2_) as a screening tool for assessment of hyperacusis, misophonia and noise sensitivity: factor analysis, validity, reliability, and minimum detectable change. *Brain Sci* 2024;15:16 39851384 10.3390/brainsci15010016 PMC11764284

[2] Swedo SE, Baguley DM, Denys D, Dixon LJ, Erfanian M, Fioretti A, et al. Consensus definition of misophonia: a delphi study. *Front Neurosci* 2022;16:841816 35368272 10.3389/fnins.2022.841816 PMC8969743

[3] Vitoratou S, Uglik-Marucha N, Hayes C, Erfanian M, Pearson O, Gregory J. Item response theory investigation of misophonia auditory triggers. *Audiol Res* 2021;11:567–81 34698077 10.3390/audiolres11040051 PMC8544191

[4] Hansen HA, Leber AB, Saygin ZM. What sound sources trigger misophonia? Not just chewing and breathing. *J Clin Psychol* 2021;77:2609–25 34115383 10.1002/jclp.23196

[5] Aryal S, Prabhu P. Understanding misophonia from an audiological perspective: a systematic review. *Eur Arch Otorhinolaryngol* 2023;280:1529–45 36484853 10.1007/s00405-022-07774-0

[6] Dixon LJ, Sevier CJ, Freshley AM. Emotion dysregulation in misophonia: findings from a nationally representative sample. *J Psychiatr Res* 2024;180:266–72 39467402 10.1016/j.jpsychires.2024.10.022

[7] Ferrer-Torres A, Giménez-Llort L. Misophonia: a systematic review of current and future trends in this emerging clinical field. *Int J Environ Res Public Health* 2022;19:6790 35682372 10.3390/ijerph19116790 PMC9180704

[8] Brennan CR, Lindberg RR, Kim G, Castro AA, Khan RA, Berenbaum H, et al. Misophonia and hearing comorbidities in a collegiate population. *Ear Hear* 2024;45:390–9 37789522 10.1097/AUD.0000000000001435

[9] Mattson SA, D’Souza J, Wojcik KD, Guzick AG, Goodman WK, Storch EA. A systematic review of treatments for misophonia. *Pers Med Psychiatry* 2023;39-40:100104 37333720 10.1016/j.pmip.2023.100104 PMC10276561

[10] Sakarya MD, Çakmak E. Mizofoni Ölçeği’nin Türkçe Formunun Geçerlik ve Güvenirlik Sınama Çalışması. *Psikoloji Çalışmaları* 2022;42:231–55

[11] Barahmand U, Stalias-Mantzikos ME, Xiang Y, Rotlevi E. The New York Misophonia Scale (NYMS): a new instrument to identify Misophonia in the general population. *J Psychiatr Pract* 2023;29:269–81 37449825 10.1097/PRA.0000000000000724

[12] Johnson M, Dozier T. *Misophonia assessment questionnaire (MAQ).* Revised by T Dozier. Livermore, CA: Misophonia Institute; 2013

[13] Rosenthal MZ, Anand D, Cassiello-Robbins C, Williams ZJ, Guetta RE, Trumbull J, et al. Development and initial validation of the duke misophonia questionnaire. *Front Psychol* 2021;12:709928 34659024 10.3389/fpsyg.2021.709928 PMC8511674

[14] Rinaldi L, Ward J, Simner J. A Factor Structure within Misophonia: The Sussex Misophonia Scale for researchers and clinicians. 2021; 10.31234/osf.io/5eb39

[15] Simner J, Rinaldi LJ, Ward J. An automated online measure for misophonia: the Sussex Misophonia Scale for adults. *Assessment* 2024;31:1598–614 38414185 10.1177/10731911241234104 PMC11528938

[16] Campbell J. Misophonia: a need for audiological diagnostic guidelines. *J Am Acad Audiol* 2023;34:176–80 37429565 10.1055/a-2125-7645 PMC11586086

[17] Erfanian M, Kartsonaki C, Keshavarz A. Misophonia and comorbid psychiatric symptoms: a preliminary study of clinical findings. *Nord J Psychiatry* 2019;73:219–28 31066600 10.1080/08039488.2019.1609086

[18] Rosenthal MZ, McMahon K, Greenleaf AS, Cassiello-Robbins C, Guetta R, Trumbull J, et al. Phenotyping misophonia: psychiatric disorders and medical health correlates. *Front Psychol* 2022;13:941898 36275232 10.3389/fpsyg.2022.941898 PMC9583952

[19] Camarata S, Miller LJ, Wallace MT. Evaluating sensory integration/sensory processing treatment: issues and analysis. *Front Integr Neurosci* 2020;14:556660 33324180 10.3389/fnint.2020.556660 PMC7726187

[20] Koziol LF, Budding DE, Chidekel D. Sensory integration, sensory processing, and sensory modulation disorders: putative functional neuroanatomic underpinnings. *Cerebellum* 2011;10:770–92 21630084 10.1007/s12311-011-0288-8

[21] Brown C, Cromwell RL, Filion D, Dunn W, Tollefson N. Sensory processing in schizophrenia: missing and avoiding information. *Schizophr Res* 2002;55:187–95 11955978 10.1016/s0920-9964(01)00255-9

[22] Brown C, Tollefson N, Dunn W, Cromwell R, Filion D. The adult sensory profile: measuring patterns of sensory processing. *Am J Occup Ther* 2001;55:75–82 11216370 10.5014/ajot.55.1.75

[23] Metz AE, Boling D, DeVore A, Holladay H, Liao JF, Vlutch KV. Dunn’s model of sensory processing: an investigation of the axes of the four-quadrant model in healthy adults. *Brain Sci* 2019;9:35 30736461 10.3390/brainsci9020035 PMC6406387

[24] Kılıç C, Öz G, Avanoğlu KB, Aksoy S. The prevalence and characteristics of misophonia in Ankara, Turkey: population-based study. *BJPsych Open* 2021;7:e144 34353403 10.1192/bjo.2021.978 PMC8358974

[25] Wu MS, Lewin AB, Murphy TK, Storch EA. Misophonia: incidence, phenomenology, and clinical correlates in an undergraduate student sample. *J Clin Psychol* 2014;70:994–1007 24752915 10.1002/jclp.22098

[26] Zhou X, Wu MS, Storch EA. Misophonia symptoms among Chinese university students: incidence, associated impairment, and clinical correlates. *J Obsessive Compuls Relat Disord* 2017;14:7–12

[27] Yektatalab S, Mohammadi A, Zarshenas L. The prevalence of misophonia and its relationship with obsessive-compulsive disorder, anxiety, and depression in undergraduate students of Shiraz University of Medical Sciences: a cross-sectional study. *Int J Community Based Nurs Midwifery* 2022;10:259–68 36274664 10.30476/IJCBNM.2022.92902.1888 PMC9579453

[28] Andermane N, Bauer M, Simner J, Ward J. A symptom network model of misophonia: from heightened sensory sensitivity to clinical comorbidity. *J Clin Psychol* 2023;79:2364–87 37341653 10.1002/jclp.23552

[29] Efraim Kaufman A, Weissman-Fogel I, Rosenthal MZ, Kaplan Neeman RK, Bar-Shalita T. Opening a window into the riddle of misophonia, sensory over-responsiveness, and pain. *Front Neurosci* 2022;16:907585 35992931 10.3389/fnins.2022.907585 PMC9381840

[30] Newman CW, Jacobson GP, Spitzer JB. Development of the tinnitus handicap inventory. *Arch Otolaryngol Head Neck Surg* 1996;122:143–8 8630207 10.1001/archotol.1996.01890140029007

[31] Khalfa S, Dubal S, Veuillet E, Perez-Diaz F, Jouvent R, Collet L. Psychometric normalization of a hyperacusis questionnaire. *ORL J Otorhinolaryngol Relat Spec* 2002;64:436–42 12499770 10.1159/000067570

[32] Erinc M, Derinsu U. Turkish adaptation of Khalfa Hyperacusis Questionnaire. *Medeni Med J* 2020;35:142–50 32733764 10.5222/MMJ.2020.97947 PMC7384510

[33] Dunn W, Brown C. Factor analysis on the sensory profile from a national sample of children without disabilities. *Am J Occup Ther* 1997;51:490–5 9242854 10.5014/ajot.51.7.490

[34] Üçgül MŞ, Karahan S, Öksüz Ç. Reliability and validity study of Turkish version of Adolescent/Adult Sensory Profile. *Br J Occup Ther* 2017;80:510–16

[35] Naylor J, Caimino C, Scutt P, Hoare DJ, Baguley DM. The prevalence and severity of misophonia in a UK undergraduate medical student population and validation of the Amsterdam Misophonia Scale. *Psychiatr Q* 2021;92:609–19 32829440 10.1007/s11126-020-09825-3 PMC8110492

[36] Paunovic KŽ, Milenković SM. The proposed criteria for high perceived misophonia in young healthy adults and the association between misophonia symptoms and noise sensitivity. *Noise Health* 2022;24:40–8 35900389 10.4103/nah.nah_40_20 PMC9703820

[37] Aryal S, Prabhu P. Misophonia: prevalence, impact and co-morbidity among Mysore University students in India - a survey. *Neurosci Res Notes* 2022;5:161

[38] Sarigedik E, Gulle BT. A study on validation of amsterdam misophonia scale in turkish and misophonia s prevalence in Turkish high school/college student population. *Psychiatry and Behavioral Sciences* 2021;11:258

[39] Pfeiffer E, Allroggen M, Sachser C. The prevalence of misophonia in a representative population-based survey in Germany. *Soc Psychiatry Psychiatr Epidemiol* 2025;60:257–64 38963546 10.1007/s00127-024-02707-0 PMC11790775

[40] Siepsiak M, Rosenthal MZ, Raj-Koziak D, Dragan W. Psychiatric and audiologic features of misophonia: use of a clinical control group with auditory over-responsivity. *J Psychosom Res* 2022;156:110777 35259551 10.1016/j.jpsychores.2022.110777

[41] Karabulut E, Çayköylü A, Özkan B. Community mental health in Türkiye and Europe: data and influencing factors. *Psikiyatride Güncel Yaklaşımlar* 2024;16:673–82

[42] Jastreboff PJ, Jastreboff MM. The neurophysiological approach to misophonia: theory and treatment. *Front Neurosci* 2023;17:895574 37034168 10.3389/fnins.2023.895574 PMC10076672

[43] Eijsker N, Schröder A, Smit DJA, van Wingen G, Denys D. Structural and functional brain abnormalities in misophonia. *Eur Neuropsychopharmacol* 2021;52:62–71 34273684 10.1016/j.euroneuro.2021.05.013

[44] Grossini E, Stecco A, Gramaglia C, De Zanet D, Cantello R, Gori B, et al. Misophonia: analysis of the neuroanatomic patterns at the basis of psychiatric symptoms and changes of the orthosympathetic/parasympathetic balance. *Front Neurosci* 2022;16:827998 36033627 10.3389/fnins.2022.827998 PMC9406292

[45] Kumar S, Tansley-Hancock O, Sedley W, Winston JS, Callaghan MF, Allen M, et al. The brain basis for misophonia. *Curr Biol* 2017;27:527–33 28162895 10.1016/j.cub.2016.12.048 PMC5321671

[46] Uddin LQ, Nomi JS, Hébert-Seropian B, Ghaziri J, Boucher O. Structure and function of the human insula. *J Clin Neurophysiol* 2017;34:300–6 28644199 10.1097/WNP.0000000000000377 PMC6032992

[47] Berger JI, Gander PE, Kumar S. A social cognition perspective on misophonia. *Philos Trans R Soc Lond B Biol Sci* 2024;379:20230257 39005025 10.1098/rstb.2023.0257 PMC11444241

[48] Mulligan S, Douglas S, Armstrong C. Characteristics of idiopathic sensory processing disorder in young children. *Front Integr Neurosci* 2021;15:647928 33994966 10.3389/fnint.2021.647928 PMC8113623

[49] Lane SJ, Mailloux Z, Schoen S, Bundy A, May-Benson TA, Parham LD, et al. Neural Foundations of Ayres Sensory Integration®. *Brain Sci* 2019;9:153 31261689 10.3390/brainsci9070153 PMC6680650

